# Closed loop stimulation helps with weaning from chronotropic incompetence-related ventilator dependence

**DOI:** 10.1007/s10840-021-01074-z

**Published:** 2021-11-18

**Authors:** Shu-I. Lin, Feng-Ching Liao, Wei-Ru Chiou, Po-Lin Lin, Jen-Yuan Kuo, Cheng-Ting Tsai, Ying-Hsiang Lee

**Affiliations:** 1 Cardiovascular Center, MacKay Memorial Hospital, 92, Zhongshan N. Rd. Sec 2, Taipei, Taiwan 10449; 2 Department of Medicine, MacKay Medical College, New Taipei, Taiwan; 3 Division of Cardiology, Taitung MacKay Memorial Hospital, Taitung, Taiwan; 4 Division of Cardiology, Hsinchu MacKay Memorial Hospital, Hsinchu, Taiwan; 5 Department of Artificial Intelligence and Medical Application, MacKay Junior College of Medicine, Nursing, and Management, Taipei, Taiwan

**Keywords:** Closed loop stimulation, Chronotropic incompetence, Heart failure, CLS, Pacemaker

A 67-year-old female patient had coronary artery disease, but her elective total revascularization did not improve the symptoms of heart failure. After 1 month, she presented with acute on chronic kidney injury and several days of respiratory distress (RD). The images and inflammatory markers such as white blood cell counts and procalcitonin excluded pneumonia and lung diseases.

**Fig. 1:**
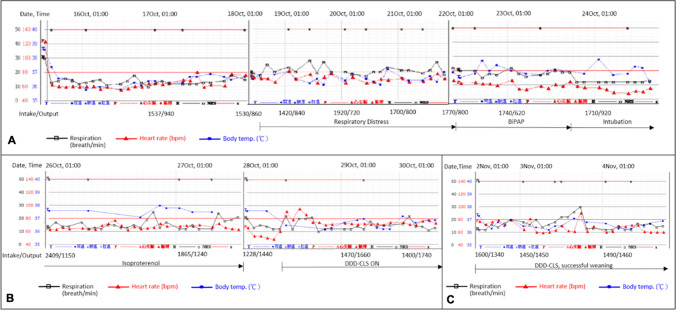
Heart rate < 70 bpm during respiratory distress and intubation, suggesting severe chronotropic incompetence. Isoproterenol testing increases pulse rate and urine output. Closed loop stimulation pacing program (DDD-CLS) facilitates weaning off the ventilator with timely breath-pulse rate correlation

Intensive diuretic regimes and non-invasive ventilation support failed to improve RD or disease status, thus necessitating invasive ventilation support (Fig. 1A). Pulmonary edema had subsided post-intensive care, but ventilation support could not be removed from the minimum pressure support ventilator. The patient was subsequently diagnosed with heart failure (HF) with a preserved ejection fraction (60%), and pulmonary capillary wedge pressure was high at 25 mmHg after 14 days of hospitalization. The other echocardiographic parameters about the valves or chambers were not relevant but > 15 of mean *E*/*E*′ ratio.

Further review revealed heart rate (HR) remained less than 70 bpm during resting stress events (e.g., RD, intubation), suggesting a diagnosis of severe chronotropic incompetence (CI) (Fig. 1A, B). This was supported by increased urine and pulse rate in isoproterenol tests (Fig. 1B).

A pacemaker equipped with HR adaptation functionality was therefore necessary. To ensure that HR response was modulated by stress rather than only motion, a closed loop stimulation (CLS) pacing program (DDD-CLS; Biotronik Evia DR-T) was chosen over a conventional rate-responsive, accelerometer-equipped sensor, or newer peak endocardial acceleration pacemaker. Minute-ventilation (MV) sensor might be another choice, but MV-mediated HR change at the beginning of stress is slower than CLS. For CI pace setting, the CLS rate was set at 60/125, “high” response, and a resting control rate of + 20 bpm. The paced and sensed AV delays were 150 and 120 ms, respectively, with IRSplus to preserve intrinsic atrioventricular nodal conduction. Following implantation, the sum of intake minus output of daily fluid turned to be negative (Fig. 1B). Moreover, the patient successfully weaned off the ventilator at 5 days post implant and avoided over-diuresis, with an HR closely associated with breath rate (Fig. 1C).

Advanced chronic HF patients (peak VO2 < 14.0 ml/kg/min) usually developed CI, which influences mortality and the incidence of major adverse cardiovascular events. CI should, therefore, be considered in resting HF status and warrants CLS for effective HR adaptation, particularly in physically inactive patients.

